# The Tumour‐Suppressive Role of miRNA‐Loaded MSC‐Derived Extracellular Vesicles in Osteosarcoma: A Systematic Review and Meta‐Analysis

**DOI:** 10.1155/tswj/5346779

**Published:** 2026-10-25

**Authors:** Izzah Munirah Hanapi, Siti Rohaliza Alitar, Mudiana Muhamad, Sharaniza Ab-Rahim

**Affiliations:** ^1^ Department of Biochemistry and Molecular Medicine Faculty of Medicine Sungai Buloh Campus Universiti Teknologi MARA Sungai Buloh Selangor Malaysia uitm.edu.my; ^2^ Institute of Medical and Molecular Biotechnology (IMMB) Faculty of Medicine Sungai Buloh Campus Universiti Teknologi MARA Sungai Buloh Selangor Malaysia uitm.edu.my

**Keywords:** exosomes, extracellular vesicles, mesenchymal stem cells, miRNA, osteosarcoma metastasis

## Abstract

Osteosarcoma (OS) is a malignant bone tumour that mostly affects adolescents. Although conventional treatment approaches improved disease prognosis significantly, high metastasis rate and recurrence impose a substantial disease burden on patients. Therefore, innovation of targeted therapy is of pressing importance towards better disease prognosis. Several in vitro studies using microRNA delivered via mesenchymal stem cell‐derived extracellular vesicles presented significant results in reducing OS metastatic properties. Henceforth, the objective of this study, which is to compile and analyse data from relevant studies, subsequently provides a comprehensive and evidence‐based summary that validates the tumour‐suppressive role of miRNA‐loaded MSC‐derived EVs targeting OS metastasis. Studies were systematically selected from three databases: PubMed, Web of Science and Scopus, according to the PRISMA criteria and flowchart. Experimental data regarding proliferation, apoptosis, migration and invasion were extracted using WebPlotDigitizer and analysed using the Comprehensive Meta‐Analysis (CMA) software. A systematic search from databases identified 33 studies, with four studies fulfilling the inclusion and exclusion criteria after final screening process. The pooled meta‐analysis demonstrated that miRNA‐loaded MSC‐derived EVs were associated with a significant decrease in proliferation, migration and invasion rates of the OS cells, with a significant increase in apoptosis rate. Moreover, low to moderate heterogeneity was observed across selected studies. This review confirms that miRNA‐loaded MSC‐derived EVs significantly suppressed OS metastasis by inhibiting proliferation, migration and invasion while promoting apoptosis. However, clinical translation is currently limited by the lack of standardised protocols necessary for large‐scale trials to validate EV‐based therapies as a potential therapeutic approach in the future.

## 1. Introduction

Osteosarcoma (OS) is a primary malignant bone tumour mainly prevalent in children and essentially localised at bone growth plates, such as the femur, tibia and humerus. Worldwide disease incidence has been reported in patients aged between 15 and 19 years old, parallel to the puberty stage [1]. Patients suffering from OS without metastasis have a 60%–70% survival rate, but once malignancy spreads to the lungs, lymph nodes and other parts of the bones, the survival rate decreases significantly [2]. In fact, the 5‐year relative survival rate for patients presented with metastasis dropped to approximately 20*%* ± 5*%* [3]. This aggressive clinical progression is attributable to mutations in tumour‐suppressor genes, such as the tumour protein p53 and retinoblastoma 1, which maintain genomic stability. These mutations remove the constraints on normal proliferation and differentiation of osteoblastic progenitors, leading to disorganised osteoid matrix [4]. Although conventional therapeutic interventions, such as neoadjuvant chemotherapy and surgical resection, provided marginally improved outcomes, high recurrence rate of nearly 30% underscores an urgent need to better understand the molecular pathways and tumour microenvironment (TME) that drive disease progression [5].

Recently, extracellular vesicles (EVs) have been the subject of intense study as key mediators of cell‐to‐cell communication, particularly regarding how microRNAs (miRNAs) within these vesicles can regulate the posttranscriptional translation and degradation of target messenger RNAs (mRNAs) to potentially inhibit oncogenic signalling [6]. The EVs carry various cargo types from their cell sources, including proteins (tetraspanins, endosomal sorting complexes required for transport [ESCRT] proteins, etc.), nucleic acids (mRNA, miRNA, etc.) and lipids (sphingomyelin, cholesterol, etc.) [7]. Hence, the cell source of EVs crucially determines their cargo and compositions, as for instance, metastatic breast cancer cells release exosomes high in miR‐105 and miR‐181c, whereas colonic cancer cells exhibit high levels of exosomal miR‐1229, miR‐21 and let‐7a [8]. A current approach, which utilises the mesenchymal stem cells (MSCs) widely known for their self‐renewal and differentiation potential, is used in regenerative medicine as a source of EVs because it has been shown to inherit their parental cell therapeutic potentials, such as homing ability and immune regulation [9]. Among MSC‐derived EVs, specific miRNAs have been reported to suppress tumour properties across multiple cancer types. These include miR‐99b‐5p targeting IGF1R (insulin‐like growth factor 1 receptor) in prostate cancer, miR‐222 downregulating ATF3 (activating transcription factor 3) in colorectal cancer and miRNA‐mediated MARCKS (myristoylated alanine‐rich C kinase substrate) inhibition in breast cancer, all of which showed reduced tumour growth, progression, or cell motility [9].

Dysregulation of miRNA expression is known to contribute to the aggressive nature of OS, including metastatic potential [10]. Therefore, correcting the miRNA dysregulation may reduce the aggressive nature of OS by inducing tumour‐suppressing pathways or inhibiting the pathways that promote invasion and metastasis. Despite the tumour‐suppressive effects of miRNA‐loaded MSC‐derived EVs in several tumour types, no study has systematically synthesised the evidence in OS to determine which specific miRNA‐loaded MSC‐derived EVs suppress tumourigenesis and metastasis. Identifying the key miRNA candidates and the factors that determine their efficacy is important to pursue clinical development in OS. Individual studies vary in miRNA identity, MSC source and OS model, and this heterogeneity prevents researchers from identifying the optimal experimental conditions. In this meta‐analysis, we compile and quantitatively analyse the evidence to identify the key determinants (miRNA, MSC source, OS model, etc) that predict therapeutic efficacy in metastatic OS.

## 2. Methods

### 2.1. Search Strategy

Relevant studies throughout the year 2018 to 2025 were obtained by searching using keywords that were constructed with Boolean operators:

• PubMed: (((“mesenchymal stem cells” OR “MSC”) AND (“exosome” OR “exosomes” OR “EV”)) AND (“miRNA” OR “microRNA” OR “miR”) AND (“invasiveness” OR “metastasis” OR “pro‐tumor” OR “apoptosis” OR “tumor suppress” OR “anti‐tumor”) AND (osteosarcoma OR osteogenic sarcoma))).
• Scopus: TITLE‐ABS‐KEY (((“mesenchymal stem cells” OR “MSC”) AND (“exosome” OR “exosomes” OR “EV”)) AND (“miRNA” OR “microRNA” OR “miR”) AND (“invasiveness” OR “metastasis” OR “pro‐tumor” OR “apoptosis” OR “tumor suppress” OR “anti‐tumor”) AND (“osteosarcoma OR osteogenic sarcoma”)).
• Web of Science: TS = ((“mesenchymal stem cells” OR “MSC”) AND (“exosome” OR “exosomes” OR “EV”)) AND (“miRNA” OR “microRNA” OR “miR”) AND (“invasiveness” OR “metastasis” OR “pro‐tumor” OR “apoptosis” OR “tumor suppress” OR “anti‐tumor”) AND (osteosarcoma OR “osteogenic sarcoma”)).

The PRISMA criteria and flow diagram were followed accordingly during the screening of the literature with the inclusion and exclusion criteria.

### 2.2. Population, Intervention, Comparison and Outcome (PICO) Principle

The PICO principle was established and listed as follows:

• Population (P): in vitro models of OS (MG63, HOS, 143B, Saos‐2 and U2OS).
• Intervention (I): treatment with EV‐derived MSCs containing miRNAs.
• Comparison (C): no treatment with EV‐derived MSCs containing miRNAs group.
• Outcome (O): antitumourigenesis effects (proliferation, apoptosis, migration and invasion).

### 2.3. Inclusion Criteria

The following is the inclusion criteria from the meta‐analysis and systematic reviews:

1. In vitro models of OS (MG63, HOS, 143B, Saos‐2 and U2OS).
2. Treatment with miRNA‐containing exosomes derived from naïve MSCs.
3. Studies that report antitumour outcome following treatment with MSC‐derived miRNA‐containing exosomes.
4. Original research articles with experimental data.
5. Published as full‐text, free‐access and peer‐reviewed articles in the English language.

We restricted inclusion to MG63, HOS, 143B, Saos‐2 and U2OS because these are the most widely used and functionally characterised human OS cells in the literature, ensuring comparability across studies. Additionally, these cell lines are most used models for studying OS metastasis in vitro. Naïve MSC‐derived EVs, which are EVs from MSCs without genetic engineering, are preferred to assess the intrinsic, endogenous tumour‐suppressive capacity of MSC‐EVs. This distinguishes our analysis from studies using engineered MSCs as delivery vehicles for a chosen cargo, which represent a different therapeutic paradigm.

### 2.4. Exclusion Criteria

The following is the exclusion criteria from the meta‐analysis and systematic reviews:

1. Studies where exosomes/EVs were derived from other types of cells.
2. Studies use cells other than OS cells.
3. Studies that use other exosomal RNA, such as long noncoding RNA (lncRNA), circular RNA (circRNA), mRNA.
4. Studies where MSC‐derived exosomes/EVs are used as a delivery vehicle for other therapeutic agents.
5. Review articles, meta‐analyses, conference abstracts, editorials and articles without experimental validation.
6. Articles with no access to full‐text articles and in a language other than English.

### 2.5. Screening Process

The authors (IMH, SAR and MMS) screened the studies obtained from the databases by their titles and abstracts. The studies were further screened by their abstracts and full text according to the inclusion and exclusion criteria as presented in the PRISMA flowchart (Figure 1). Any disagreements were resolved in discussion with all the contributing authors.

**Figure 1:**
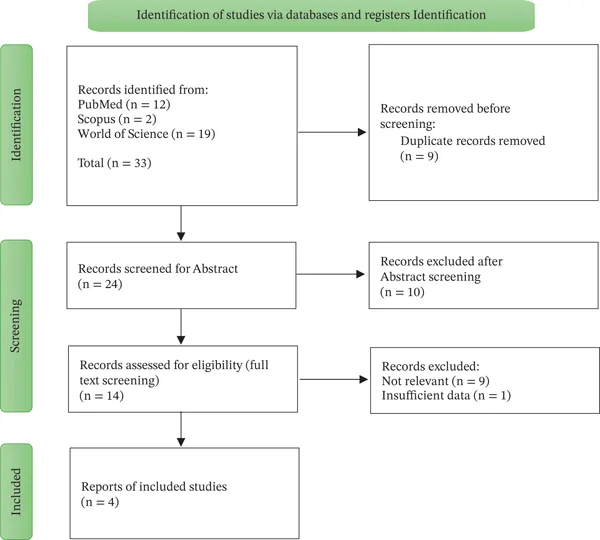
PRISMA flow diagram to systematically identify studies investigating MSC‐derived EVs delivering miRNA in OS cells.

### 2.6. Quality Assessment

Risk of bias was assessed using the SYRCLE risk of bias tool for animal studies, modified to fit for evaluating in vitro studies. Currently, there is no universally accepted risk of bias tool for in vitro model. Unsuitable domains in SYRCLE risk of bias tool were removed and added domains relevant to cell‐based assays. The domains “allocation concealment” and “random housing” were removed, as they are inapplicable to cell culture experiments. “Sequence generation” was rephrased as “Randomization” to assess random allocation of experimental units (wells, plates). “Baseline characteristics” was rephrased as “Baseline Comparability” to assess comparability of cell passage number, seeding density and reagent batch between groups. Blinding items were retained but reframed for the in vitro context: “Blinding of Operators” (personnel applying interventions) and “Blinding of Assessors” (personnel measuring outcomes). We added two domains: (1) Replication and independence, assessing whether experiments were independently repeated (biological replicates) versus technical replicates only; and (2) other sources of bias, capturing unit‐of‐analysis errors, contamination and inappropriate controls. The “Random Order of Assessment” domain was retained to assess whether samples were processed in random order to avoid instrument drift bias. The assessment was done by IMH and SRA, and any conflicts were settled through deliberation between the two authors.

### 2.7. Data Extraction

Each selected literature was carefully examined to extract the data collected in the data extraction sheets. The data extraction sheets were separated into five categories: study identification, intervention and model details, outcome data extraction, data extracted from graphs and mechanistic data and pathways. Mean and standard deviation (SD) were extracted from graphs of each selected experiment using WebPlotDigitizer.

### 2.8. Statistical Analysis

The meta‐analysis for each experiment was performed through the Comprehensive Meta‐Analysis (CMA) software to determine the correlation between the treatment (intervention) and the control group. The effect‐size metric was Hedges′ g, which is appropriate for small sample sizes (*n* < 20 per group) and continuous outcomes. Hedges′ g corrects for bias in standardised mean differences (Cohen′s d) that occurs with small samples. A random‐effects model was prespecified for all meta‐analyses, given the anticipated methodological and biological heterogeneity across studies.

Each outcome (proliferation, migration, invasion, apoptosis) was analysed in separate meta‐analyses. In order to avoid unit of analysis error, each study contributed only one effect size per outcome. For Xu et al. (2020), U2OS and HOS data were reported; only HOS dataset was used to generate meta‐analysis for all outcomes. Xu et al. (2020) and Ruan et al. (2024) reported several time points for proliferation outcome, which only 48 h dataset was used to generate the proliferation outcome. Where a study reported multiple assays for the same outcome construct (e.g., wound healing and transwell assays for migration), these were analysed as separate outcomes because they capture different biological processes. Forest plots were generated with 95% confidence intervals (CI).

Heterogeneity was assessed using Cochran′s *Q* test and the *I* ^2^ statistic. *I* ^2^ values of 25%, 50% and 75% were interpreted as low, moderate and high heterogeneity, respectively. A *p* value < 0.10 for Cochran′s Q was considered indicative of statistically significant heterogeneity. Sensitivity analysis was performed by removing one study individually and recalculating the estimated pooled effect estimate each time.

## 3. Results

### 3.1. Search Outcomes and Study Parameters

Database search from PubMed, Scopus and Web of Science results in 33 studies. After removing duplicates, studies (*n* = 24) were screened for their title and abstract, excluding 10 studies from the pool. Full text screening was performed, and a total of four studies remained with relevant information and sufficient data. The PRISMA flowchart (Figure 1) shows the methodical screening process of the studies. The OS cell line used in the study, OS cell origin, cellular properties, sample size, culture conditions and control groups are presented in Table 1. The studies used metastatic OS cell lines to establish their experimental model, where one study used both U2OS and HOS cell lines, another study used MG63 and the remaining two used the 143B cell line.

**Table 1:** Summary of in vitro model establishment from each study.

Study	OS cell line	OS cell origin	Cellular properties	Sample size (biological replicates)	Culture conditions	Control group
Xu et al., 2020	U2OS, HOS	Human	Metastatic	*n* = 3	Standard 2D	Mock group (untransfected), MSC‐Exo‐67 group (negative control)
Ruan et al., 2024	MG63	Human	Metastatic	*n* = 3	Standard 2D	MG63 group (untransfected)
Zhang et al., 2020	143B	Human	Highly metastatic	*n* = 3	Standard 2D	NC group (negative control)
Keremu et al., 2022	143B	Human	Highly metastatic	*n* = 3	Standard 2D	Blank (untransfected), NC group (negative control—PBS)

*Note:* 2D denotes the standard monolayer culture.

The parameters of the miRNA‐containing MSC‐derived exosome treatment, with details on MSC source, EV isolation method, EV concentration used in treatment, treatment duration with EV, cargo miRNA and miRNA modifications, are compiled in Table 2. All studies used human‐sourced MSC and ultracentrifugation as the EV isolation method. Two studies [11, 12] did not specify the dose of EVs utilised in the treatment. One study [13] set the dosage at 50 *μ*g/mL, whereas another study [14] used twice the dosage (100 *μ*g/mL) in the treatment. As for treatment duration, one study [13] did not report the specific treatment duration, whereas two studies [11, 12] reported the duration set at 48 h, and the remaining one study [14] was set at 24 h. In terms of miRNA, two studies [12, 14] chose miR‐206 as the cargo miRNA in the EVs, whereas one used miR‐150 [11] and another utilised miR‐22 [13] in their study. The overall results and effects of miRNA‐loaded MSC‐derived EVs on the OS cells are summarised in Table 3.

**Table 2:** Mesenchymal stem cell (MSC)‐derived exosome treatment parameters summarised from each study.

Study	MSC source	EV isolation method	EV dosage	Treatment duration with EV	Cargo miRNA	miRNA modifications
Xu et al., 2020	Human—tissue not specified	Ultracentrifugation	NS^a^	48 h	miR‐150	Overexpression miR‐150 in EVs
Ruan et al., 2024	Human—tissue not specified	Ultracentrifugation	50 *μ*g/mL	NS^a^	miR‐22	Native expression miR‐22 in EVs
Zhang et al., 2020	Human bone marrow	Ultracentrifugation	NS^a^	48 h	miR‐206	Overexpression miR‐206
Keremu et al., 2022	Human bone	Ultracentrifugation	100 *μ*g/mL	24 h	miR‐206	Native expression miR‐206 in EVs

^a^NS, not specified in original manuscript.

**Table 3:** Primary suppressive outcomes in selected experiments in selected studies.

Study	Assay method	Assay outcome type	Assay results	Molecular target/pathway
Xu et al., 2020	MTT assay	Proliferation rate	Proliferation rate ↓	Target: IGF2BP1 Pathway: tumour suppressive pathway
	Flow cytometry	Apoptosis (%)	Apoptosis ↑	
	Wound healing assay	Migration rate (%)	Migration rate ↓	
	Transwell analysis	Invasion rate (%)	Invasion rate ↓	
Ruan et al., 2024	CCK‐8 assay	Proliferation rate (%)	Proliferation rate ↓	Target: Twist1 Pathway: EMT‐related tumour suppressive pathway
	Wound healing assay	Migration rate (%)	Migration rate ↓	
	Transwell assay (migration)	Number of migrated cells	Number of migrated cells ↓	
	Transwell assay (invasion)	Number of invaded cells	Number of invaded cells ↓	
Zhang et al., 2020	EdU assay	Proliferation rate (%)	Proliferation rate ↓	Target: TRA2B Pathway: EMT‐related tumour suppressive pathway
	Flow cytometry	Apoptosis rate (%)	Apoptosis rate ↑	
	Wound healing assay	Width of injured line (%)	Migration rate ↓	
	Transwell assay (migration)	Number of migrated cells	Migration rate ↓	
	Transwell assay (invasion)	Number of invaded cells	Invasion rate ↓	
Keremu et al., 2022	EdU assay	Proliferation rate (%)	Proliferation rate ↓	Target: NRSN2 Pathway: ERK1/2‐Bcl‐xL signalling pathway
	Flow cytometry	Apoptosis rate (%)	Apoptosis rate ↑	
	Transwell assay (migration)	Number of migrated cells	Migration rate ↓	
	Transwell assay (invasion)	Number of invaded cells	Invasion rate ↓	

*Note:* The symbol “↑” indicates increased rate; “↓” indicates decreased rate.

### 3.2. Proliferation Rate

All four studies have investigated the proliferation rates of the OS cells after treatment with the miRNA‐loaded MSC‐derived EVs. Meta‐analysis assessment regarding the proliferation rate was done, and a significant decrease (Hedges^′^ g = −5.993, 95*%*CI = –7.827, –4.159, overall Z − value = –6.404) in the proliferation rate between the treatment group and control group can be observed in Figure 2. Pooled CI for proliferation as in Table 4, is narrow due to the zero between‐study heterogeneity (*I* ^2^ = 0.000, Q − value (chi − square) = 1.136) for the outcome, which causes the random effects model to approximate a fixed effect model. As this analysis was based on only four studies, the pooled effect should be viewed as preliminary, and its precision and applicability to broader contexts remain uncertain.

**Table 4:** Basic statistics of meta‐analysis results for the effect of miRNA‐loaded MSC‐derived EVs on the proliferation rate of the OS cell line.

	Effect size and 95% confidence interval						Test of null	
Model	Number of studies	Point estimate	Standard error	Variance	Lower limit	Upper limit	Z‐value	*p*
Random	4	−5.993	0.936	0.876	−7.827	−4.159	−6.404	0.000

*Note:* Heterogeneity: *Q* = 1.136, *d* *f* (*Q*) = 3, *p* = 0.768, *I* ^2^ = 0.000, Tau^2^ = 0.000.

**Figure 2:**
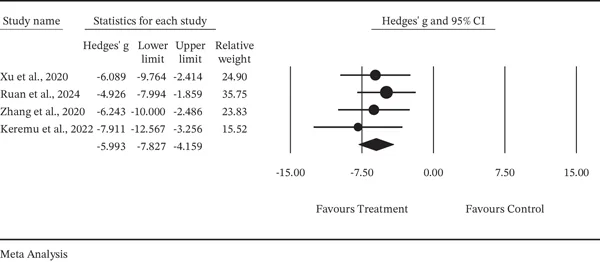
Forest plot for proliferation rate of OS cells after treatment with MSC‐derived EVs.

### 3.3. Apoptosis Rate

Three of four studies participated in the investigation of the apoptosis rate after the miRNA‐loaded MSC‐derived EV treatment. The results demonstrated a significant increase in the rate of apoptosis in OS cells after EV treatment compared with the control group (Hedges^′^ g = 7.033, 95*%*CI = 2.620, 11.446, overall Z − value = 3.123) in Figure 3. The results of the heterogeneity test on the apoptosis rate among the studies (Table 5) show that the studies have substantial heterogeneity (*I* ^2^ = 65.663, Q − value (chi − square) = 5.825). The result should be considered exploratory. The limited number of studies contributing to this outcome (n = 3) warrants cautious interpretation, as both the reliability of the effect estimate, and its generalisability are constrained. The substantial heterogeneity further limits the precision of this estimate, and the result should be considered exploratory.

**Figure 3:**
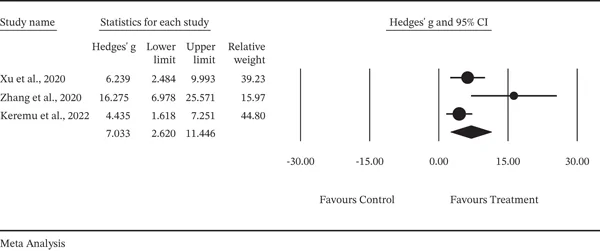
Forest plot for apoptosis rate of OS cells after treatment with MSC‐derived EVs.

**Table 5:** Basic statistics of meta‐analysis results for the effect of miRNA‐loaded MSC‐derived EVs on the apoptosis rate of the OS cell line.

	Effect size and 95% confidence interval						Test of null	
Model	Number of studies	Point estimate	Standard error	Variance	Lower limit	Upper limit	Z‐value	*p*
Random	3	7.033	2.252	5.070	2.620	11.446	3.123	0.002

*Note:* Heterogeneity: *Q* = 5.825, *d* *f* (*Q*) = 2, *p* = 0.054, *I* ^2^ = 65.663, Tau^2^ = 9.253.

### 3.4. Migration Rate (Wound Healing Assay)

Three studies used wound healing assays to measure the migration rate in a collective migration manner. The meta‐analysis result in Figure 4 presents a significant decrease in the collection migration rate (Hedges^′^ g = –3.828, 95*%*CI = –5.307, –2.348, Z − value = –5.070). A random effects model was used in the heterogeneity test presented in Table 6, which showed a low degree of heterogeneity (*I* ^2^ = 0.000, Q − value = 1.317). With only three studies informing this pooled estimate, the result is provisional; its precision and external validity should not be overestimated.

**Figure 4:**
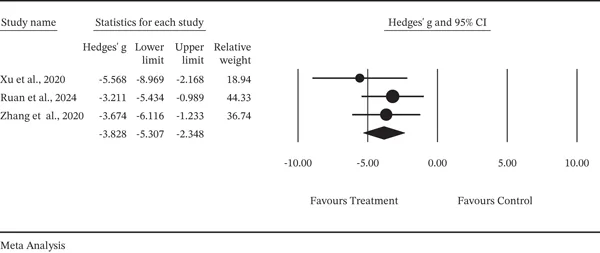
Forest plot for migration rate on wound healing assay of OS cells after treatment with MSC‐derived EVs.

**Table 6:** Basic statistics of meta‐analysis results for the effect of miRNA‐loaded MSC‐derived EVs on the migration rate (wound healing assay) of the OS cell line.

	Effect size and 95% confidence interval						Test of null	
Model	Number of studies	Point estimate	Standard error	Variance	Lower limit	Upper limit	Z‐value	*p*
Random	3	−3.828	0.755	0.570	−5.307	−2.348	−5.070	0.000

*Note:* Heterogeneity: *Q* = 1.317, *d* *f* (*Q*) = 2, *p* = 0.518, *I* ^2^ = 0.000, Tau^2^ = 0.000.

### 3.5. Migration Rate (Transwell Assay)

The transwell assay demonstrates cell migration in a stimulated manner and measures the 3D migration through a membrane. Figure 5 presents three of the pooled studies analysed in meta‐analysis, which results in a significant decrease in the migration rate between the group treated with miRNA‐loaded MSC‐derived EVs and the control group (Hedges^′^ g = –5.888, 95*%*CI = –7.996, –3.781, Z − value = –5.476). The heterogeneity test in Table 7 shows a low degree of heterogeneity among the studies (*I* ^2^ = 0.000, Q − value = 1.582). Given the small evidence base of three studies in the meta‐analysis, this pooled estimate carries substantial uncertainty regarding both its precision and its relevance beyond the specific experimental conditions studied.

**Figure 5:**
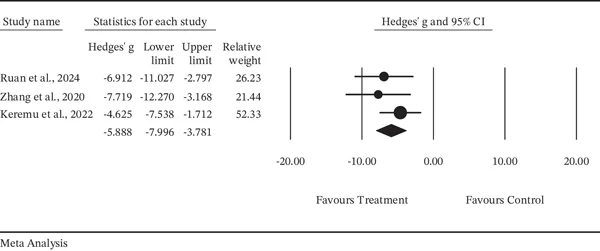
Forest plot for migration rate on transwell assay of OS cells after treatment with MSC‐derived EVs.

**Table 7:** Basic statistics of meta‐analysis results for the effect of miRNA‐loaded MSC‐derived EVs on the migration rate (transwell assay) of the OS cell line.

	Effect size and 95% confidence interval						Test of null	
Model	Number of studies	Point estimate	Standard error	Variance	Lower limit	Upper limit	Z‐value	*p*
Random	3	−5.888	1.075	1.156	−7.996	−3.781	−5.476	0.000

*Note:* Heterogeneity: *Q* = 1.582, *d* *f* (*Q*) = 2, *p* = 0.453, *I* ^2^ = 0.000, Tau^2^ = 0.000.

### 3.6. Invasion Rate

All selected studies investigated the invasion rate after the exposure of miRNA‐containing EVs to the OS cells, as shown in Figure 6. A significant decrease in invasion rate was discovered in all four studies (Hedges^′^ g = –5.144, 95*%*CI = –7.502, –2.786, Z − value = –4.275). The heterogeneity test presented in Table 8 showed moderate heterogeneity between the involved studies (*I* ^2^ = 49.426, Q − value = 5.932). The small number of studies (*n* = 4) limits the confidence that can be placed in this estimate, and readers should interpret both its precision and generalisability with caution. The moderate heterogeneity suggests some variability across studies, and the estimate should be interpreted with caution.

**Figure 6:**
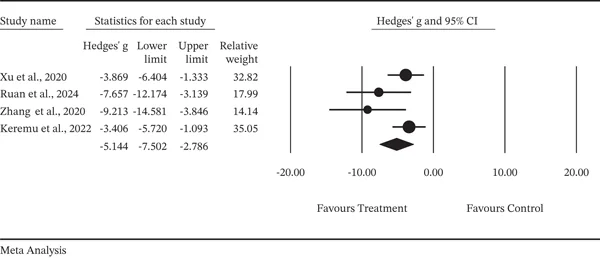
Forest plot for invasion rate of OS cells after treatment with MSC‐derived EVs.

**Table 8:** Basic statistics of meta‐analysis results for the effect of miRNA‐loaded MSC‐derived EVs on the invasion rate of the OS cell line.

	Effect size and 95% confidence interval						Test of null	
Model	Number of studies	Point estimate	Standard error	Variance	Lower limit	Upper limit	Z‐value	*p*
Random	4	−5.144	1.203	1.448	−7.502	−2.786	−4.275	0.000

*Note:* Heterogeneity: *Q* = 5.932, *d* *f* (*Q*) = 3, *p* = 0.115, *I* ^2^ = 49.426, Tau^2^ = 2.737.

### 3.7. Sensitivity Test

Sensitivity analysis for proliferation showed in Figure 7 that the pooled effect size ranged from −5.640 (95% CI: −7.636 to −3.645) when Keremu et al. was removed to −6.586 (95% CI: −8.874 to −4.298) when Ruan et al. was removed. The original pooled estimate (−5.993, 95% CI: −7.827 to −4.159) fell within this range, and statistical significance was maintained across all iterations, indicating that the overall result was consistent to the exclusion of any single study.

**Figure 7:**
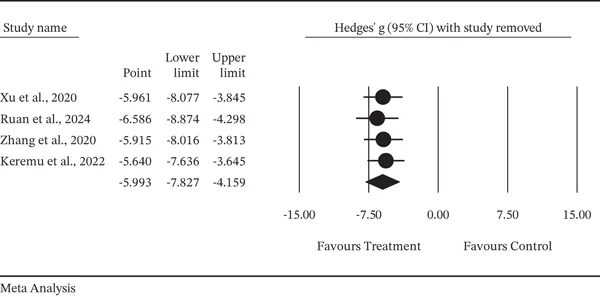
Sensitivity analysis for the proliferation rate of OS cells after treatment with miRNA‐loaded MSC‐derived EVs. Each row shows the pooled Hedges′ g and 95% confidence interval recalculated with one study removed.

Sensitivity analysis for apoptosis in Figure 8 revealed that the pooled effect was not stable across leave‐one‐out iterations, ranging from 5.084 (95% CI: 2.831–7.337) to 10.319 (95% CI: 0.657–19.980). Notably, exclusion of Xu et al. resulted in a pooled estimate of 9.492 with a CI that crossed zero (−1.988 to 20.971), rendering the result nonsignificant. This fragility highlights the influence of individual studies on the apoptosis estimate.

**Figure 8:**
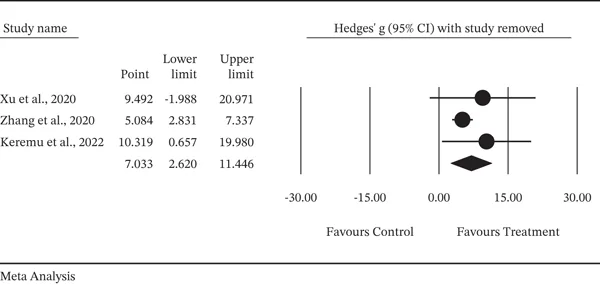
Sensitivity analysis for the apoptosis rate of OS cells after treatment with miRNA‐loaded MSC‐derived EVs. Each row shows the pooled Hedges′ g and 95% confidence interval recalculated with one study removed.

Sensitivity analysis for migration (wound healing assay) shown in Figure 9 and the pooled effect size ranged from –3.421 (95% CI: –5.065 to –1.777) when Xu et al. was removed to –4.319 (95% CI: –6.302 to –2.335) when Ruan et al. was removed. The original pooled estimate (–3.828, 95% CI: –5.307 to –2.348) fell within this range, and statistical significance was maintained across all iterations, indicating that the overall result was robust to the exclusion of any single study.

**Figure 9:**
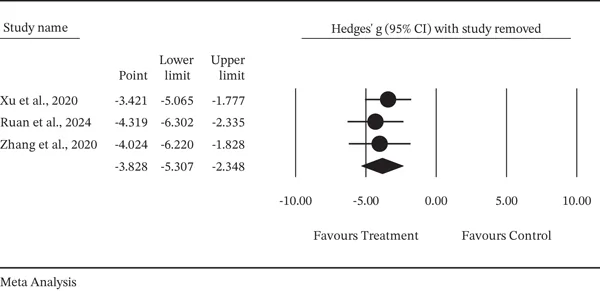
Sensitivity analysis for migration rate (wound healing assay) of OS cells after treatment with miRNA‐loaded MSC‐derived EVs. Each row shows the pooled Hedges′ g and 95% confidence interval recalculated with one study removed.

Sensitivity analysis for migration (transwell assay) in Figure 10 showed that the pooled effect size ranged from –5.389 (95% CI: –7.766 to –3.011) when Zhang et al. was removed to –7.275 (95% CI: –10.327 to –4.223) when Keremu et al. was removed. The original pooled estimate (–5.888, 95% CI: –7.996 to –3.781) fell within this range, and statistical significance was maintained across all iterations, indicating that the overall result was maintained to the exclusion of any single study.

**Figure 10:**
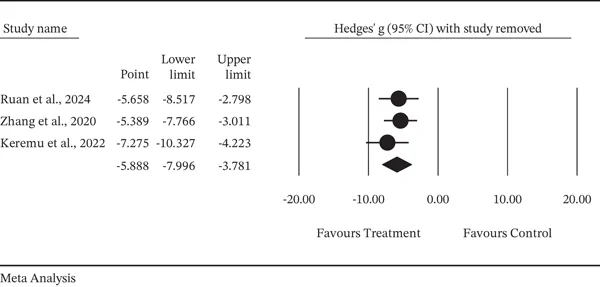
Sensitivity analysis for migration rate (transwell assay) of OS cells after treatment with miRNA‐loaded MSC‐derived EVs. Each row shows the pooled Hedges′ g and 95% confidence interval recalculated with one study removed.

Sensitivity analysis for invasion is shown in Figure 11 and the pooled effect size ranged from –4.275 (95% CI: –6.228 to –2.321) when Zhang et al. was removed to –6.291 (95% CI: –9.692 to –2.890) when Keremu et al. was removed. The original pooled estimate (–5.144, 95% CI: –7.502 to –2.786) fell within this range, and statistical significance was maintained across all iterations, indicating that the overall result was consistent to the exclusion of any single study.

**Figure 11:**
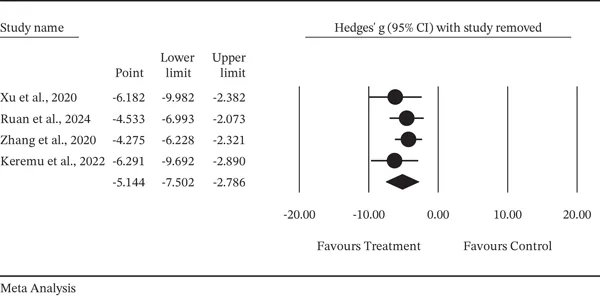
Sensitivity analysis for invasion rate of OS cells after treatment with miRNA‐loaded MSC‐derived EVs. Each row shows the pooled Hedges′ g and 95% confidence interval recalculated with one study removed.

### 3.8. Risk of Bias Results

All selected studies were evaluated for risk of bias. However, due to the nature of in vitro studies, none of the articles mentioned randomisation or blinding of assessors/operators. The questionnaire′s overall results revealed that the articles have “some concerns,” where one or more key domains have been evaluated as “unclear risk,” but none of those key domains have “high risk.”

## 4. Discussion

This review presents the results of meta‐analyses evaluating the suppressive role of MSC‐derived EVs loaded with miRNA towards OS metastasis in vitro. The selected studies reported that the exposure of the miRNA‐loaded MSC‐derived EVs to OS cells including proliferation, survival, migration and invasion. As EVs mediate cell–cell communication in the TME and can promote tumour growth and metastasis [15], MSC‐derived EVs transporting tumour‐suppressing miRNAs have high potential to be included and intervene in the metastatic cascade.

The pooled analysis revealed a significant decrease in OS cell proliferation following treatment with miRNA‐loaded MSC‐derived EVs, with no heterogeneity across the four studies, indicating mutual agreement despite differences in proliferation assays, EV isolation methods, miRNA cargo and cell lines. The CI does not cross zero, indicating statistical significance. However, with only four studies, this estimate should be interpreted cautiously and does not yet establish clinical reliability. For apoptosis, the significant positive increase in effect size indicates that miRNA‐loaded MSC‐derived EVs promote OS cell death, though substantial heterogeneity observed for apoptosis (*I* ^2^ = 65.7*%*) may be attributable to differences in miRNA cargo (miR‐150 vs. miR‐206), EV dosage (not specified vs. 100 *μ*g/mL), treatment duration (24 h vs. 48 h) and OS cell line (HOS vs. 143B). Notably, the two studies using miR‐206 (Zhang et al., 2020; Keremu et al., 2022) reported widely differing effect sizes (Hedges^′^ g = 16.275 vs. 4.435), despite sharing the same miRNA cargo and cell line, suggesting that EV dosage or treatment duration may be the dominant moderator. The study using miR‐150 (Xu et al., 2020) reported an intermediate effect (Hedges^′^ g = 6.239). Given the small number of studies, formal subgroup analysis was not statistically powered, but these observations highlight the need for standardised reporting of EV dosage and treatment duration in future studies. Migration was assessed via wound healing (collective 2D migration and gap closure, influenced by cell proliferation and speed) and transwell assays (directional chemotactic movement through a porous membrane). Both methods resulted in a negative pooled effect size, indicating reduced migration rate across experimental formats. This suggests that the antimigratory response is consistent across different experimental formats. However, with only three studies per analysis, this finding requires confirmation in larger datasets. The invasion rate was evaluated using transwell assay with Matrigel coating, showing a significant decrease and confirming reduced invasive ability of OS cells. Moderate heterogeneity was observed for invasion (*I* ^2^ = 49.4*%*). The two studies using miR‐206 reported divergent effect sizes (Zhang et al., 2020: Hedges^′^ g = −9.213; Keremu et al., 2022: Hedges^′^ g = −3.406), despite using the same cell line (143B). This divergence may reflect differences in EV dosage (not specified vs. 100 *μ*g/mL) and treatment duration (48 h vs. 24 h). The study using miR‐22 (Ruan et al., 2024) reported a large effect (Hedges^′^ g = −7.657) in MG63 cells, while the study using miR‐150 (Xu et al., 2020) reported a more modest effect (Hedges^′^ g = −3.869) in HOS cells. These patterns suggest that miRNA cargo, EV dosage and cell line baseline invasiveness may all contribute to heterogeneity.

This review purposely focused on MSC‐derived EVs as delivery vehicles for therapeutic miRNAs. MSCs are multipotent stem cells with high self‐renewal and differentiation abilities such as trans‐differentiation and immune regulation, making them promising candidates for cell‐based therapies in regenerative medicine [16]. In contrast to their therapeutic abilities, MSCs are also active participants in the TME, contributing to tumour development and promoting the metastatic cascade [17]. MSC‐derived EVs inherit the similar tumour homing capability of their cell source, which makes them suitable vehicles to target tumour cells [17, 18]. This homing ability enables the EVs to migrate to the TME of the primary or metastatic site and deliver the miRNA to inhibit tumour progression.

miRNAs are short, noncoding RNA strands involved in the posttranscription modifications of mRNAs. The binding of miRNA to the 3 ^′^‐UTR of a specific mRNA results in the degradation or the inhibition of mRNA translation [19]. The delivery of miRNA disrupts the expression of tumour genes, subsequently dysregulating a specific molecular pathway, thus presenting a targeted tumour approach. Articles pooled in this study used bioinformatic databases or gene expression experiments to determine the downregulated miRNAs in OS, deeming that the restoration of expression would exert antitumour effects. Xu et al. (2020) reported that miR‐150 expression was restored, and metastatic properties were decreased by binding to the IGF2BP1 mRNA, which caused negative regulation of the protein. Ruan et al. (2024) reported that miR‐22 expression, which targeted Twist1, subsequently downregulated its protein expression involved in the EMT‐related tumour suppressive pathway. Other studies used miR‐206 targeting the OS metastasis. Zhang et al. (2020) documented that OS development was associated with the downregulation of miR‐206 and the upregulation of TRA2B protein. The study demonstrated that miR‐206 targeted the TRA2B mRNA, inhibiting its translation. As a result, the TRA2B protein was downregulated, which affected proteins involved in the EMT pathway, such as Twist, N‐cadherin and E‐cadherin. Meanwhile, Keremu et al. (2022) reported a potential targeting interaction between miR‐206 and NRSN2 using the results from the Starbase database and dual‐luciferase assay. Moreover, the overexpression of miR‐206 via MSC‐derived EV delivery was shown to suppress OS cell proliferation, invasion and migration, as well as promote apoptosis, which is attributed to the translational inhibition of NRSN2 mRNA. Ultimately, this ability of a single miRNA to target multiple distinct molecules and disrupt unrelated pathways demonstrates a highly efficient “one‐to‐many” therapeutic model, providing a more comprehensive blockade of tumour progression than conventional single‐target treatments.

MSC‐derived EVs with miRNA cargo effectiveness in suppressing metastasis in OS are still under research. Various miRNAs and tumour types have varying profiles of miRNA, requiring initial investigation to identify suitable miRNAs and targets to specify the treatment parameters to induce their tumour suppressive effects. EV dosage and treatment duration are also critical as EVs have a limited half‐life in the body [20]. Furthermore, the potential for using engineered EVs as delivery vehicles for synergistic therapies, such as combining conventional chemotherapeutics with bone morphogenetic proteins, warrants investigation to overcome multidrug resistance observed in the OS stroma [21]. Standardising EV isolation and storage protocols is ultimately crucial since any differences in the methods and conditions can significantly impact the biological integrity and safety profile of the EV cargo before application in clinical practice [22]. Consequently, further research is vital to refine the mechanisms of the delivery, as MSC‐derived EVs with miRNA cargo hold significant promise as a more beneficial and potentially less invasive, preliminary therapeutic strategy for managing OS.

## 5. Limitations of This Study

This meta‐analysis has several limitations that should be considered when interpreting the findings. The pooled effect sizes observed in this meta‐analysis are larger than those typically reported in clinical studies. This is consistent with the controlled conditions of in vitro experiments and the low within‐study variability (*n* = 3 biological replicates per group). These effect sizes should not be directly extrapolated to clinical settings. The small number of included studies (3–4 per outcome) limits the precision and generalisability of the pooled estimates. The narrow CI observed for some outcomes reflect low between‐study heterogeneity, which may be imprecisely estimated with few studies. These findings should be considered preliminary and require validation in larger, independent studies before clinical translation.

The included studies also differed in several key parameters, which may contribute to heterogeneity and limit comparability. Three different miRNAs were used: miR‐150, miR‐22 and miR‐206, each targeting a distinct mRNA and operating through a different molecular pathway. Pooling these studies assumes a shared underlying mechanism, which may not be valid. The MSC source was inconsistently reported: two studies did not specify the tissue of origin (Xu et al., 2020; Ruan et al., 2024), one used bone marrow (Zhang et al., 2020) and one used bone (Keremu et al., 2022). MSCs from different tissues may release EVs with different cargo profiles. EV dose was also inconsistent: two studies did not report the dose (Xu et al., 2020; Zhang et al., 2020), one used 50 *μ*g/mL (Ruan et al., 2024) and one used 100 *μ*g/mL (Keremu et al., 2022). EV isolation protocols may have differed in centrifugation speeds and durations. Additionally, different OS cell lines were used: HOS (Xu et al., 2020), MG63 (Ruan et al., 2024), and 143B (Zhang et al., 2020; Keremu et al., 2022), which differ in baseline metastatic potential and genetic background. Because the same studies that used miR‐206 also used 143B, the effects of miRNA type and cell line are perfectly confounded and cannot be separated.

Data extraction from published graphs using WebPlotDigitizer introduces a further potential source of error. Although this software is widely used in meta‐analyses, extracted values may differ slightly from original raw data due to limited resolution or inconsistent axis calibration. Minor discrepancies cannot be fully excluded.

Formal assessment of publication bias was not performed because the small number of included studies (*n* = 3–4 per outcome) provides insufficient statistical power to reliably detect funnel plot asymmetry or to yield a meaningful Egger′s test result. Nonetheless, publication bias favouring positive findings remains likely in this field and cannot be excluded.

## Author Contributions

Sharaniza Ab‐Rahim and Izzah Munirah Hanapi contributed to the study conception and design. The literature search and study screening were performed by Izzah Munirah Hanapi, Sharaniza Ab‐Rahim and Mudiana Muhamad. Quality assessment and data extraction were conducted by Izzah Munirah Hanapi and Siti Rohaliza Alitar. Data analysis was performed by Izzah Munirah Hanapi. The first draft of the manuscript was written by Izzah Munirah Hanapi.

## Funding

This study was supported by the Ministry of Higher Education, Malaysia (10.13039/501100003093; FRGS/1/2024/SKK10/UITM/02/6).

## Disclosure

All authors critically revised and approved the final manuscript.

## Conflicts of Interest

The authors declare no conflicts of interest.

## Data Availability

The data that support the findings of this study are available from the corresponding author upon reasonable request.
